# Aberrant Hematopoiesis and Morbidity in Extremely Preterm Infants With Intrauterine Growth Restriction

**DOI:** 10.3389/fped.2021.728607

**Published:** 2021-11-12

**Authors:** Nora J. Reibel, Christof Dame, Christoph Bührer, Tobias Muehlbacher

**Affiliations:** ^1^Department of Neonatology, Charité - Universitätsmedizin Berlin, Berlin, Germany; ^2^Department of Neonatology, University Hospital Zurich, Zurich, Switzerland

**Keywords:** anemia, coagulation disorder, neutropenia, sepsis, thrombocytopenia

## Abstract

**Background and Objective:** Intrauterine growth restriction (IUGR) poses additional challenges in extremely low gestational age newborns (ELGANs). We assessed disturbed hematopoiesis and morbidities associated with this disorder.

**Methods:** This single-center retrospective case–control study compared perinatal hematological profiles, major morbidities, and mortality of 49 infants (gestational age <28 weeks, birth weight ≤ 3rd percentile, and compromised placental function) and 98 infants (birth weight >10th percentile) matched for gestational age, year, and sex.

**Results:** IUGR-ELGANs had significantly elevated nucleated red blood cells and lower neutrophil and platelet counts at birth and on the third day of life. During the first week of life, IUGR-ELGANs received more red blood cell, platelet, and plasma transfusions and were more intensively treated with antibiotics. Rates of infections acquired during the first week (59.2 vs. 17.3%, *p* < 0.001), severe bronchopulmonary dysplasia or death (42.9 vs. 17.3%, *p* < 0.01), and mortality (36.7 vs. 7.1%, *p* < 0.001) were markedly elevated in IUGR-ELGANs, but not of hemorrhages or other morbidities.

**Conclusions:** IUGR-ELGANs have high rates of acquired infections during the first week of life and display severe pulmonary morbidity leading to bronchopulmonary dysplasia or death. The high rate of transfusions observed in these infants warrants further scrutiny.

## Introduction

Approximately 5% of all pregnancies are associated with intrauterine growth restriction (IUGR), a condition in which the fetus is unable to reach its own biologically determined growth potential (1, 2). Higher incidences are observed in preterm infants (3, 4). IUGR is associated with increased fetal and neonatal morbidity and mortality (5). The most common underlying cause is a dysfunction of the utero-placental unit leading to the inadequate supply of oxygen and nutrients to the developing fetus (2). Chronic oxygen deprivation in the fetus triggers the synthesis of erythropoietin (EPO) resulting in the stimulation of erythropoiesis and an increased number of erythroid precursor cells (6). Subsequently to excessive erythropoiesis, neonates born with IUGR often suffer from early-onset thrombocytopenia and leucopenia (7, 8). The exact mechanisms of the complex hematopoietic disorder are still unclear. One hypothesis is that the excessively stimulated erythropoiesis causes subsequent suppression of platelet (PLT) and white blood cell (WBC) production as pluripotent precursor cells are being shifted in the direction of erythropoiesis (7–9). The hematological disorders might increase susceptibility to early-onset infections (EOI) (10) and bleeding disorders, such as intraventricular hemorrhage (IVH) and pulmonal hemorrhage (PH), particularly in very preterm infants. Concerning major bleedings, it is discussed that plasmatic coagulation disorders could be a bigger issue than thrombocytopenia (11–13).

Transfusions in the non-bleeding patient as well as prophylactic antibiotic treatment are commonly given in order to prevent these complications, despite missing evidence. In addition, two recently published randomized clinical trials (RCTs) showed an association between the administration of platelet transfusions and major hemorrhage as well as death (14, 15).

The primary aim was to investigate to which extent hematologic disorders in IUGR-ELGANs (extremely low gestational age newborns) are associated with a higher risk of major morbidity as infection, hemorrhage, bronchopulmonary dysplasia (BPD), necrotizing enterocolitis (NEC), or retinopathy of prematurity (ROP). The secondary aim was to analyze underlying causes for mortality in these high-risk infants.

## Methods

This retrospective case–control study applied a very strict definition of the study population not just as small-for gestational age (GA), but also with confirmed compromised placental function to gain robust results on the hematologic disorders and the associated sequelae. Therefore, we identified all very low birth weight (VLBW) infants with IUGR at our tertiary neonatal intensive care unit between January 2008 and December 2017 *via* the electronic digital patient management system. Eligible patients fulfilled the following inclusion criteria: GA <28 weeks, severe IUGR with birth weight ≤3rd percentile, and proof of restricted placental perfusion. The latter was defined as at least one of the following Doppler-sonographic or histopathologic criteria: Pulsatility index of the uterine artery >95th percentile (16), cerebro-placental index <1 (defined as ratio pulsatility index middle cerebral artery to pulsatility index of the umbilical artery) indicating the brain-sparing effect in infants with chronic intrauterine compromise (17), or histopathology of the placenta with proof of utero-placental dysfunction (18). Infants with major congenital malformations or chromosomal abnormalities or receiving primary palliative care were excluded. The control group was carefully selected from infants with birth weight >10th percentile to match the IUGR group as precisely as possible by (a) sex, (b) GA (±6 days), and (c) birth within ±1 year to exclude changes in treatment protocols during the study period. To strengthen the power of our analysis in a small cohort, we allocated two controls for each IUGR-ELGAN.

Data extracted from medical records and laboratory files included clinical data of pre- and perinatal care, blood parameters, mortality, neonatal morbidities, and treatment. Routinely, blood specimens were taken directly after birth from peripheral venous puncture or through an umbilical catheter to determine the values of the complete blood cell count (CBC), laboratory inflammatory parameters [interleukin-6 (IL-6) and C-reactive protein (CrP)], and, during the study period, a coagulation screen [international normalized ratio (INR), activated partial thromboplastin time (aPTT), and fibrinogen] prior to the the standard parenteral administration of 0.5 mg vitamin K. CBC was determined by using fully automated cell counters Sysmex XE-2100 and subsequently Sysmex XN10-1 (TOA Medical Electronics). CrP and IL-6 were measured with the Cobas® analyzer series (Roche) and coagulation screen with the automated coagulation analyzer Sysmex® CS-2500 and subsequently CS-5100 System (Siemens). Disorders in hematopoiesis were assessed by hemoglobin levels, nucleated red blood cells (NRBC), PLT counts, and WBC at birth. We defined a cutoff of ≥8 NRBC/nl according to the GA-adjusted percentiles by Christensen et al. (19). Thrombocytopenia in very preterm infants was defined by a PLT count <150/nl and classified into severe (0–50/nl) and moderate (51–100/nl) (20). Neutropenia was defined as an absolute neutrophil count (ANC) <1,000/μl and classified into severe (0–500/μl) or moderate (501–999/μl) (21). Abnormal coagulation screens were defined depending on both GA and the respective laboratory method (22). Neonatal infection was defined as elevated inflammatory parameters with IL-6 plasma concentrations >100 ng/L and/or CrP plasma concentrations >10 mg/L (23, 24). Chorioamnionitis was defined as elevated inflammatory parameters in the initial blood sample at primary care and proven by histopathology (25). Depending on the age at onset of infection, EOI (elevated inflammatory parameters within the first 72 hours of life), infections within the early neonatal period (I-ENP) of the first 7 days of life, and late-onset infections (LOI) (elevated inflammatory parameters after the first week) were distinguished (26). In addition, the rate of positive blood cultures was analyzed, considering that the rate is often false negative in very preterm infants due to a low blood volume transferred to the culture bottle or because of intrapartum antibiotic prophylaxis (27, 28). Rationale to distinguish between the two different periods for EOI and I-ENP was to analyze the effect of a diminished granulopoiesis on the incidence of infections. Furthermore, we analyzed antibiotic treatment until discharge, considering the duration and substance. Standard treatment protocol for chorioamnionitis and EOI were ampicillin plus gentamicin during the whole study period, while the treatment protocol of I-ENP and LOI changed from piperacillin plus cefotaxime to ampicillin/sulbactam plus gentamicin in 2011. Suspected or proven catheter-associated infections were treated with ampicillin/sulbactam plus vancomycin, and suspected or proven meningitis was additionally treated with cefotaxime. In case of life-threatening infections, treatment was escalated to second-line antibiotics as vancomycin plus meropenem. Discontinuation of antibiotic treatment depended on negative results of inflammatory parameters and clinical improvement. In case of culture-positive sepsis or meningitis, treatment was continued for at least 7 days.

The following interventions were recorded: Transfusions of red blood cells (RBC), PLT, and fresh frozen plasma (FFP) during the first week (volume and day of any transfusion), administration of exogenous surfactant, duration of mechanical ventilation, oxygen (O_2_) supplementation, and inhaled nitric oxide.

Outcome variables were mortality prior to discharge and major morbidities: IVH severity was graded as by Papile (29). PH was defined by the presence of frank blood from the trachea, which required prompt intervention as an increased respiratory support or RBC transfusions, as well as a multi-lobular opacity on chest x-rays (30). Persistent pulmonary hypertension (PPHN) was diagnosed by transthoracic echocardiography with a flattening of the interventricular septum and right ventricular systolic pressure (measured by tricuspid regurgitation) at least equal systemic systolic pressure or right to left shunt *via* the ductus arteriosus (31). NEC was defined according to Bell et al. (32), while spontaneous intestinal perforation (SIP) was diagnosed by explorative laparotomy, which is standard care in our institution in case of radiologic evidence of intestinal perforation. The classification of ROP was done according to the international classification (33). BPD was rated according to the National Institute of Health classification (34).

The study was approved by the Institutional Review Board (EA2/053/18).

### Statistical Analysis

Statistical comparisons were performed with SPSS for Windows 26.0 (SPSS, Inc., Chicago, Illinois). For analysis of continuous variables, we used Mann–Whitney *U*-test, and categorical variables were compared by two-sided Fisher's exact test. *p*-values < 0.05 were regarded to be of statistical significance. Binary logistic regression analysis was performed to examine independent factors for mortality, EOI, I-ENP, LOI, prevalence of hemorrhagic events, and BPD. A Kaplan–Meier curve was used to illustrate mortality during the neonatal period.

## Results

**Figure 1:**
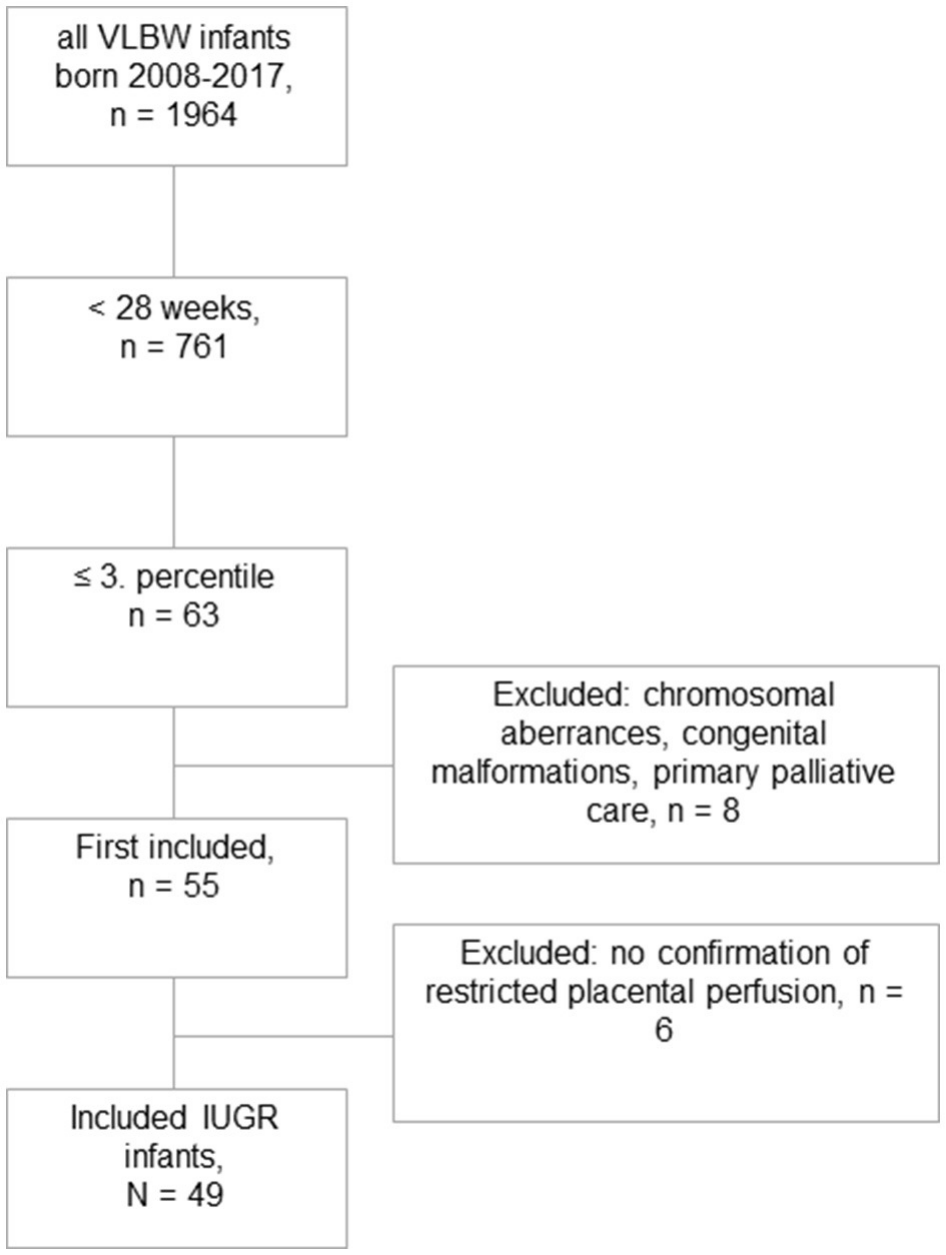
Flowchart of this retrospective case–control study. Out of 1,964 very low birth weight born infants during the study period, 49 could finally be included with severe IUGR ≤ 3rd percentile and evidence of uteroplacental dysfunction.

Among a total number of 135 preterm infants with a GA <28 weeks and a birth weight below the 10th percentile, we identified 63 infants with severe IUGR (birth weight ≤3rd percentile), out of which eight received primary palliative care or were excluded due to major congenital malformations or chromosomal abnormalities. Another six infants were excluded due to missing signs of restricted placental perfusion (Figure 1). The remaining eligible 49 infants were matched by GA, sex, and year of birth in a 1:2 fashion to 98 controls. The groups of IUGR-ELGANs and controls significantly differed by birth weight, mode of delivery, and umbilical artery pH but not Apgar scores (Table 1).

**Table 1:** Demographic and clinical data of the ELGANs with severe IUGR (birth weight ≤3rd percentile) and the age-matched control group.

	**Severe IUGR (*n* = 49)**	**Controls (*n* = 98)**	***p*-value**
**Birth weight (g)**	430 (270–570)	820 (514–1245)	<0.001
**Gestational age (weeks)**	25.6 (23.4–27.6)	25.7 (22.9–27.7)	0.69
**Female**	22 (44.9)	44 (44.9)	1.0
**Singletons**	45 (91.8)	73 (74.5)	<0.05
**Pulsatility index umbilical artery**	1.92 (0.94–3.30)	1.09 (0.69–1.73)	<0.001
**Cerebro-Placental-Ratio < 1**	31/37 (83.8)	5/26 (19.0)	<0.001
**histopathology: uteroplacental dysfunction**	34/34 (100%)	2/69 (2.9%)	<0.001
**Premature rupture of membranes**	4 (8.2)	26/92 (26.5)	<0.01
**Chorioamnionitis (histopathology and elevated inflammation parameter)**	0/34	17/69 (24.6%)	<0.001
**Antenatal steroids**	39/47 (83.0)	90/97 (92.8)	0.09
**Cesarean section**	49/49 (100)	82/98 (83.7)	<0.01
Primary	26 (53.1)	13/94 (13.8)	<0.001
**Umbilical cord artery pH**	7.27 (7.01–7.37)	7.30 (6.88–7.46)	<0.01
**Apgar score at 5 min**	7 (1-9)	7 (1-9)	0.91
5-min Apgar score <5	7 (14.3)	8 (8.2)	0.26
**Apgar score at 10 min**	8 (5-9)	8 (1-9)	0.41
10-min Apgar score < 5	0 (0)	3 (3.1)	0.55
**CRIB Score**	8 (7-16)	5 (1-14)	<0.001

*Categorical data are given as n (%), continuous data as median (range)*.

### Aberrant Hematopoiesis

CBC numbers taken at primary care indicated excessively stimulated erythropoiesis with significantly higher number of NRBC in IUGR-ELGANs, despite almost identical hemoglobin concentration. PLT count and fibrinogen concentration were lower in severe IUGR; however, the incidence of severe thrombocytopenia or abnormal coagulation screens did not differ between both groups. WBC, ANC, and count of immature granulocytes in IUGR-ELGANs were lower at birth and with higher rates of severe neutropenia. The complete data are shown in Table 2.

**Table 2:** Blood cell counts, coagulation screens, and inflammatory markers on admission.

	**Severe IUGR (*n* = 49)**	**Controls (*n* = 98)**	***p*-value**
**Red blood cells**			
Hemoglobin (g/dl)	14.6 (7.7–18)	15.1 (3.9–22.8)	0.46
NRBC (/nl)	11.2 (0.3–75.5)	2.4 (0.15–38.0)	<0.001
NRBC ≥ 8/nl	30/47 (63.8)	19/94 (20.2)	<0.001
**Platelets**			
PLT count (/nl)	115 (32–232)	220 (58–479)	<0.001
<150/nl	36/49 (73.5)	13/98 (13.3)	<0.001
<100/nl	17 (34.7)	4 (4.1)	<0.001
<50/nl	2 (4.1)	0 (0)	0.11
**Coagulation screen**			
INR	1.58 (1.09–6.51)	1.59 (1.07–5.27)	0.60
aPTT (s)	60 (42–130)	65 (19–240)	0.13
aPTT > 158 s	0/45 (0)	2/81 (2.5)	0.54
Fibrinogen (mg/dl)	98 (60–704)	143 (60–546)	<0.001
Fibrinogen < 70 mg/dl	6/44 (13.6)	4/83 (4.8)	0.09
**White blood cells**			
WBC count (/nl)	4.6 (1.7–14.4)	9.4 (3.2–63.8)	<0.001
ANC (/μl)	522 (46–3,395)	2,817 (103–40,716)	<0.001
<1000/μl	38/47 (80.9)	19/93 (20.4)	<0.001
<500/μl	22/47 (44.9)	7/93 (7.5)	<0.001
Immature granulocytes (/μl)	0 (0–521)	258 (0–15,948)	<0.001
**Inflammatory parameters**			
IL-6 (ng/L)	27 (0–304)	65.2 (0–50,000)	<0.01
CrP (mg/dl)	0.3 (0–3.3)	0.3 (0–19.4)	0.14

*In addition to absolute numbers, standard cutoff values defining severity of cytopenias are considered. Categorical data are given as n (%), continuous data as median (range)*.

On the third day of life, PLT, ANC, and immature granulocytes decreased further with significantly higher rates of severe thrombocytopenia and severe neutropenia (Table 3).

**Table 3:** This table shows PLT and WBC counts on the third day of life.

	**Severe IUGR (*n* = 49)**	**Controls (*n* = 98)**	***p*-value**
**Platelets**			
PLT count (/nl)	73 (29–200)	197 (83–498)	<0.001
<150/nl	36 (73.5)	4 (4.1)	<0.001
<50/nl	8 (16.3)	0 (0)	<0.001
**White blood cells**			
WBC count (/nl)	4.6 (1.7–14.4)	9.4 (3.2–63.8)	<0.001
ANC (/μl)	1,320 (136–5,222)	5,856 (904–62,437)	<0.001
<500/μl	9/34 (26.5)	0/54 (0.0)	<0.001
Immature granulocytes (/μl)	131 (0–1,027)	345 (0–13,998)	<0.001

*In addition to absolute numbers, standard cutoff values defining severity of cytopenias are considered. Categorical data are given as n (%), continuous data as median (range)*.

### Differences in Management and Interventions

**Figure 2:**
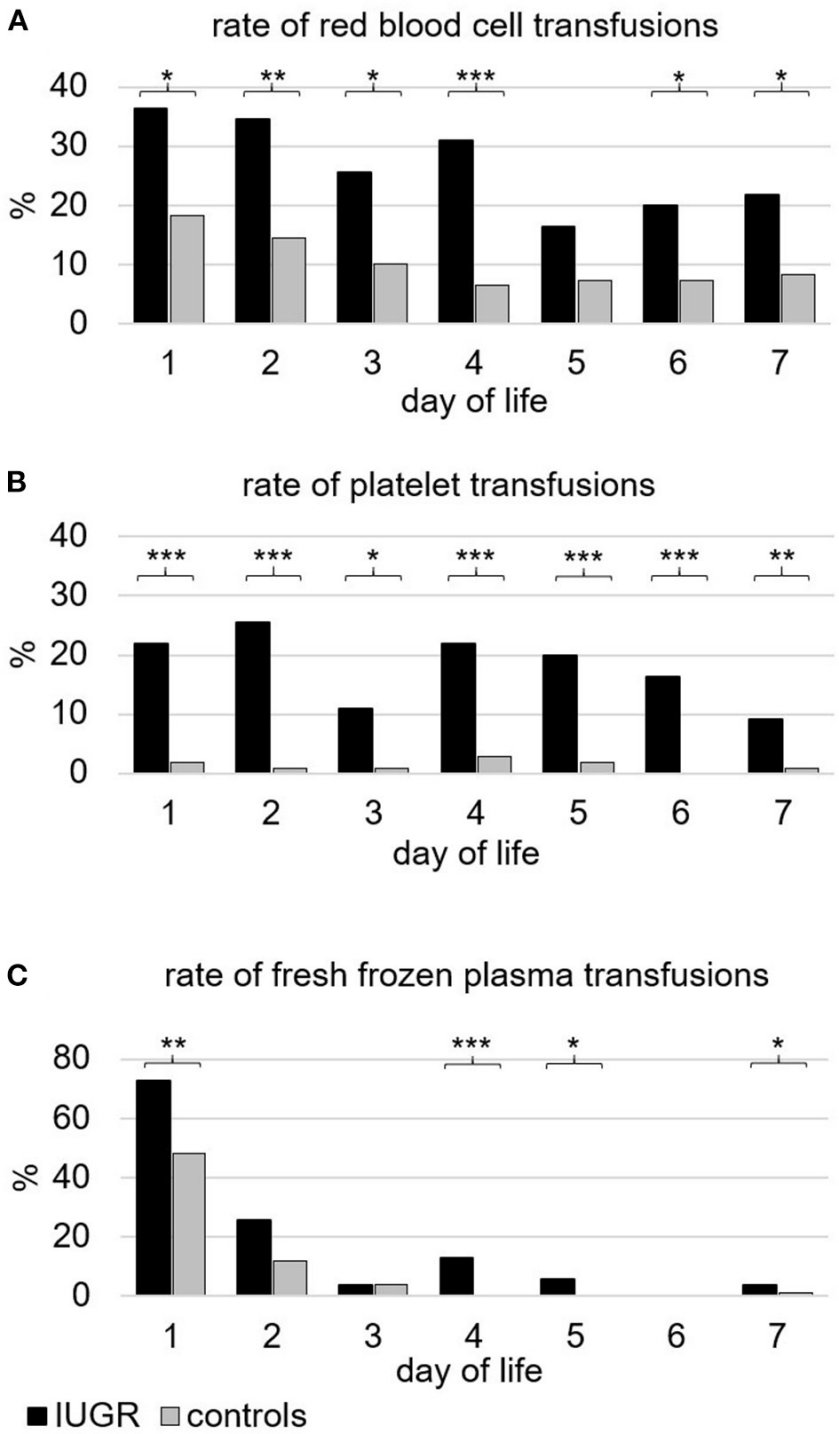
Frequency of infants (%) transfused with different blood components during the first week of life. **(A)** Packed red blood cells. In total, 83.7% of infants with severe IUGR vs. 52.0% of controls (*p* < 0.001) received one or more transfusions of red blood cells. **(B)** (Apheresis) platelet concentrates. In total, 63.3% of infants with severe IUGR vs. 6.1% of controls (*p* < 0.001) received one or more transfusions of platelets. **(C)** Fresh frozen plasma. In total, 77.6% of infants with severe IUGR vs. 58.2% of controls (*p* < 0.01) received one or more transfusions of fresh frozen plasma. Black bars indicate the groups with severe IUGR, gray bars represent the control group. *p*-values: *p < 0.05; ** *p* < 0.01; *** *p* < 0.001, two-sided Fisher exact test.

During the first week of life, the percentage of infants who received transfusions of RBC, PLT, or FFP and the respective total volume per kilogram were higher in IUGR-ELGANs compared to controls (Table 4). Figure 2 illustrates the pattern of transfusion of the respective blood component during the first 7 days after birth, indicating a decreased frequency of RBC transfusion after day 4, rather constant transfusion rates of PLTs until the end of the first week, and a majority of FFP transfusions on day 1. Rates of repeated transfusions of all blood products were higher in IUGR-ELGANs (Table 4, Supplementary Figure 1). None of the infants in our study received intrauterine RBC transfusions or had an alloimmunization requiring exchange transfusions.

**Table 4:** Treatment of ELGANs.

	**Severe IUGR (*n* = 49)**	**Controls (*n* = 98)**	***p*-value**
**Transfusions**			
RBC (ml/kg)	33.7 (0–164.5)	12.2 (0–77.1)	<0.001
PLT (ml/kg)	21.3 (0–102.8)	0.0 (0–81.4)	<0.001
FFP (ml/kg)	20.3 (0–96.8)	14.7 (0–71.4)	<0.001
Rate of repeated RBC	27 (55.1)	15 (15.3)	<0.001
Rate of repeated PLT	19 (38.8)	1 (1.0)	<0.001
Rate of repeated FFP	18 (36.7)	2 (2.0)	<0.001
**Antibiotic treatment**			
Days of antibiotic treatment	15 (0–92)	8 (0–88)	<0.001
Antibiotic treatment onday 7 of life	23/43 (65.1)	24/98 (24.5)	<0.001
Relative duration ofantibiotic treatment until day 7 of life	271/321 (84.4)	399/686 (58.2)	<0.001
Second-line antibiotics	32 (65.3)	22 (22.4)	<0.001
Escalation tosecond-line antibioticsor antibiotic reserve	22 (44.9)	13 (13.3)	<0.001
**Ventilation**			
Days on invasiveventilation	29/34 (85.3)	43/93 (46.2)	<0.001
Percent days on invasiveventilation/days until deathor discharge	30.4 (0–100)	6.8 (0–100)	<0.001
Doses of surfactant	2 (0–3)	1 (0–3)	<0.01
Days on supplementalOxygen	60 (1–263)	43.5 (0–160)	0.06
Percentage days onsupplemental oxygen/daysuntil death or discharge	100 (1.2–100)	59.1 (0–100)	<0.001

*Transfusions of blood components during the first week of life, antibiotic treatment, and respiratory support are given as median and range. Rates of repeated transfusions during the first week are given as n, (%)*.

IUGR-ELGANs received longer antibiotic treatment, more often second-line antibiotics as vancomycin and/or meropenem, and an already initiated antibiotic treatment was more often escalated to second-line antibiotics (Table 4, Supplementary Figure 2).

IUGR-ELGAN needed more respiratory support with mechanical ventilation and longer oxygen supplementation relative to days of survival or until discharge from the hospital and received more doses of surfactant (Table 4).

### Neonatal Morbidities

#### Infections

In IUGR-ELGANs, chorioamnionitis occurred less often, whereas the rates of EOI as well as I-ENP and LOI were significantly higher. The odds ratio (OR) for EOI was 5.46 (95% CI, 2.34–12.73), the OR for I-ENP was 6.83 (95% CI, 3.14–14.89), and the OR for the occurrence of at least one LOI was 2.63 (95% CI, 1.29–5.41) in IUGR-ELGANs compared to controls (Table 5). During the first 72 h of life, 40.8% of IUGR-ELGANs had elevated inflammation parameters compared to 11.2% in the control group (*p* < 0.001). Although the rates of acquired infections subsequently decreased in both groups between days 4 and 7, IUGR-ELGANs still significantly more often suffered from infections (18.4 vs. 6.1%, *p* < 0.05) (Table 5). As shown in Supplementary Figure 2 in each group, 77.6% of infants received primary antibiotic treatment, although only 39.5% of infants with severe IUGR and 56.6% of the control group met the laboratory criteria of infection on the first day (*p* < 0.05). However, in both groups, all blood culture examinations taken during the first 3 days of life remained sterile after 5 days of incubation (Table 5). Within the first week of life, IUGR remained the only independent variable (OR 6.18; 95% CI, 2.49–15.33; *p* < 0.001) for increased inflammatory markers as analyzed by binary logistic regression (variables included GA, IUGR, WBC, and ANC at birth).

**Table 5:** Neonatal morbidities and mortality.

	**Severe IUGR (*n* = 49)**	**Controls (*n* = 98)**	***p*-value**
**Inflammation**			
Elevated inflammatory parameter at birth	10 (20.4)	38 (38.7)	<0.05
EOI	20 (40.8)	11 (11.2)	<0.001
Acquired infection DOL 4 to 7	9 (18.4%)	6 (6.1%)	<0.05
I-ENP	29 (59.2)	17 (17.3)	<0.001
Positive blood culture	2/48 (4.2)	1/92 (1.1)	1.0
≥1 LOI	33 (67.3)	41 (41.8)	<0.01
Positive blood culture	18/108 (16.7)	22/102 (21.6)	0.39
Coagulase negative *staphylococci*	13/18 (72.2)	14/22 (63.6)	0.73
Pneumonia	2/49 (4.1)	6/98 (6.1)	0.72
**Hemorrhagic events**			
IVH, all grades	7 (14.3)	14 (14.3)	1.0
IVH, > grade 2	5 (10.2)	5 (5.1)	0.30
PH	2 (4.1)	2 (2.0)	0.60
**ROP**			
All stages	29/34 (85.3)	43/93 (46.2)	<0.001
≥stage 2	9/34 (26.5)	15/93 (16.1)	0.42
Intervention	7/34 (20.6)	8/93 (8.6)	0.12
**Abdominal morbidity**			
SIP	4 (8.2)	2 (2.0)	0.10
NEC	3 (6.1)	4 (4.1)	0.69
**Pulmonary morbidity**			
PPHN	11 (22.4)	3 (3.1)	<0.001
BPD_36_ or death	48 (98.0)	64 (65.3)	<0.001
Severe BPD or death	21 (42.9)	17 (17.3)	<0.01
O_2_ requirement at 36weeks postmenstrual age	21/34 (61.8)	22/91 (24.2)	<0.001
Late PAH	10/35 (28.6)	8/91 (8.8)	<0.001
Severe pulmonarymorbidity	20 (40.8)	15 (15.3)	<0.01
**Death**	18 (36.7)	7 (7.1)	<0.001

*Severe pulmonary morbidity is defined as PH or PPHN or severe BPD or PAH. Variables are given as n (%). DOL = day of life, EOI = early-onset infection (<72 h), I-ENP = infection in the early neonatal period (first 7 days), LOI = late-onset infection (>1 week of life)*.

#### Hemorrhage

As measures for the implication of thrombocytopenia and coagulation disorders, the incidences of IVH (all grades), severe IVH (> grade 2), PH, and combined major hemorrhage (defined as severe IVH or PH) were evaluated but did not differ between IUGR-ELGANs and controls (Table 5). In each group, one infant had both IVH and PH (Table 5). Using binary regression analysis (including the following variables: IUGR, GA, PLT counts at birth and at third day of life and volume of PLT transfusions), the only independent risk factor for hemorrhage remained GA (OR 0.58; 95% CI, 0.37–0.91; *p* = 0.02).

### Pulmonary Morbidity

PPHN occurred more often in IUGR-ELGANs. The incidence of moderate to severe BPD at 36 weeks of postmenstrual age, the rate of the composite outcome BPD36 or death, and late pulmonary arterial hypertension (PAH) were significantly higher in IUGR-ELGANS than in controls (Table 5). A major pulmonary morbidity (defined as PH or PPHN or development of severe BPD or PAH) was associated with IUGR with an OR of 3.82 (95% CI, 1.73–8.42). In a binary logistic regression (including GA, IUGR, duration of mechanical ventilation, total number of infections, and frequency of transfusion of RBCs, PLTs, or FFP), only GA (OR 0.36; 95% CI, 0.18–0.72; *p* < 0.05), IUGR (OR 13.57; 95% CI, 0.1.38–133.77; *p* < 0.05), and duration of mechanical ventilation (OR 1.10; 95% CI, 1.00–1.22; *p* < 0.05) remained independent factors for BPD and BPD or death.

### Major Abdominal Morbidities

NEC and SIP were not statistically different between both groups (Table 5).

### ROP

ROP (all stages) occurred more often in IUGR-ELGANS than in controls, but neither the rate of ROP ≥ stage 2 nor the need for intervention were higher (Table 5).

### Mortality

**Figure 3:**
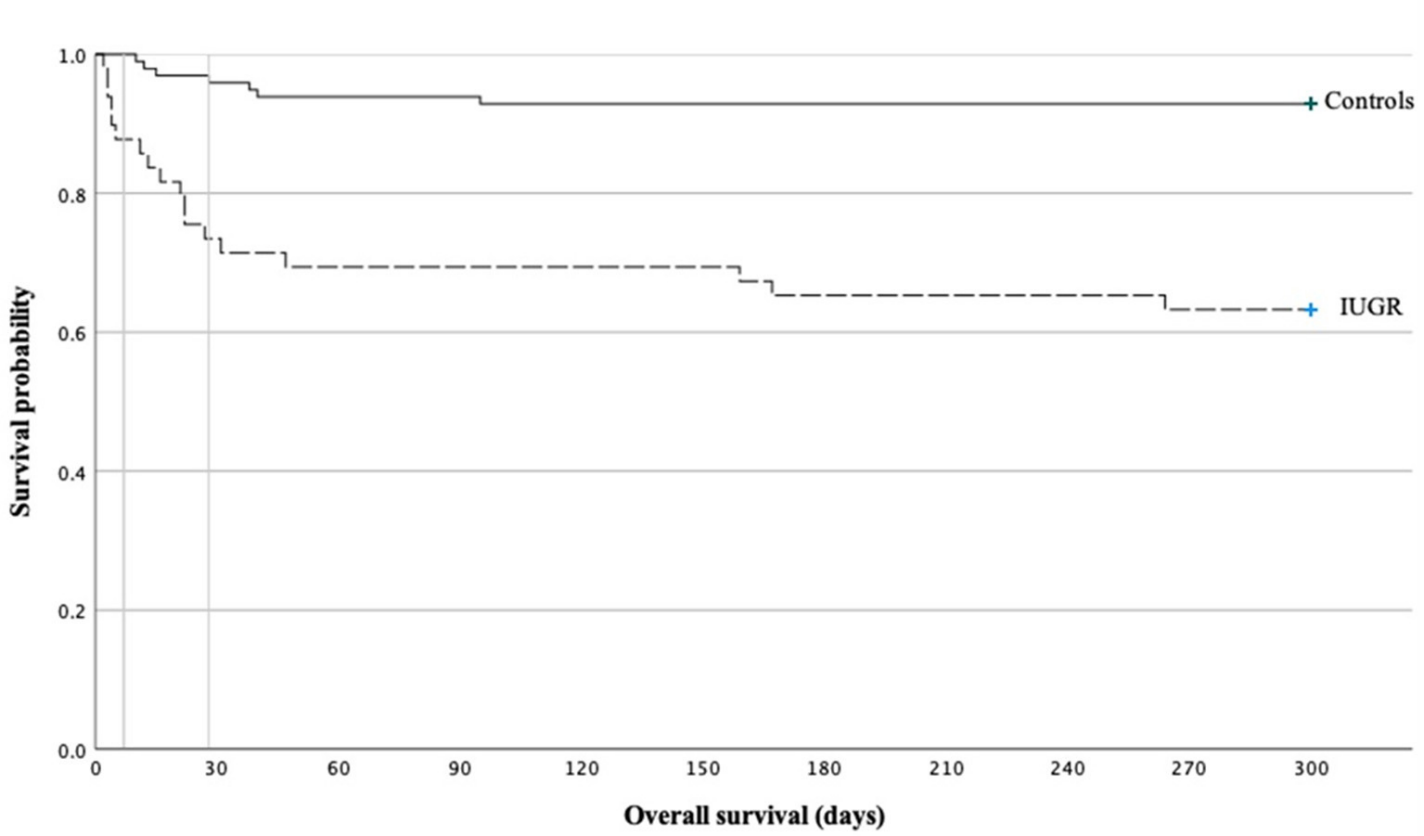
Kaplan–Meier curve on the mortality of ELGAN with severe IUGR vs. controls.

The mortality rate prior to discharge was significantly higher in IUGR-ELGANs (Figure 3) with an OR of 7.55 (95% CI, 2.88–19.78) and IUGR (OR 6.95; 95% CI, 1.98–24.42; *p* = 0.002) remained together with GA (OR 0.42; 95% CI, 0.24–0.73; *p* = 0.002) the only independent variable in a binary logistic regression (variables included GA, IUGR, severe pulmonary morbidity, severe abdominal complication, and I-ENP). Supplementary Table 1 summarizes hematologic profile, comorbidities, and interventions for deceased infants and survivors in both groups, respectively. In the early neonatal period, sepsis and PPHN were the main causes of death (Table 6 and Supplementary Table 1) in IUGR-ELGANs, yet without reaching significance level, while severe BPD with PAH caused late mortality (*p* < 0.01). In the control group, mortality was associated with LOI and major abdominal morbidity. In both groups, deceased infants were invasively ventilated longer and received more frequently and longer supplemental oxygen, antibiotic treatment (relative to days of survival or until discharge), and more RBC transfusions. Furthermore, deceased IUGR infants received more PLT transfusions (Supplementary Table 1).

**Table 6:** Causes of mortality in infants with severe IUGR and controls.

	**Severe IUGR (*n* = 18)**	**Controls (*n* = 7)**
Neonatal Infections, *n* (%)	4 (22.2)	2 (28.6)
Sepsis	4 (22.2)	1 (14.3)
Pneumonia	0	2 (28.6)
Abdominal Complications	4 (22.2)	3 (50.0)
NEC	1 (5.6)	1 (14.3)
SIP	1 (5.6)	0 (0.0)
Others	2 (11.1)	2 (28.6)
Lung Diseases/Complications	8 (44.4)	0 (0)
RDS	1 (5.6)	0 (0)
PPHN	2 (11.1)	0 (0)
BPD + PAH	5 (27.8)	0 (0)
Hemorrhagic events	1 (5.6)	0 (0)
IVH	1 (5.6)	0 (0)
Other	1 (5.6)	1 (14.3)

*Categorical data are given as n (%)*.

### Association of Disturbed Hematopoiesis and Major Morbidities in the Subgroup of IUGR-ELGANs

In binary logistic regression analysis of disturbed hematopoiesis (NRBC ≥8/nl, severe thrombocytopenia at birth or on the third day of life, ANC <500/μl, GA, birth weight), only GA was an independent variable for mortality in IUGR-ELGANS (OR 0.28; 95% CI, 0.09–0.933; *p* < 0.05).

## Discussion

### Hematological Disorders

Our retrospective cohort study confirms the high incidence of early-onset thrombocytopenia (73.5%) and neutropenia (80.9%) in ELGANs with severe IUGR. Increased numbers of NRBC may indicate that chronic fetal hypoxia shifted hematopoiesis toward erythropoiesis at the expense of megakaryopoiesis and granulopoiesis (35, 36). Although both early-onset thrombocytopenia and neutropenia due to maternal or placental factors are normally mild to moderate and self-limiting within the first week of life (36, 37), we aimed to analyze the consequences on the transfusions of donor blood components and antibiotics.

### Anemia and Red Blood Cell Transfusions

Excessive stimulation of erythropoiesis was indicated by massively elevated absolute count of NRBCs. Immediately after birth, the number of NRBC directly correlates with the intrauterine distress and is known to be associated with poor neonatal outcomes (38, 39). Notably, initial hemoglobin concentrations were very similar in IUGR-ELGANs and controls (Table 2), suggesting a shortened life span of RBCs after stress erythropoiesis (40). The higher frequency and volume of RBC transfusion during the first week of life, however, might indicate a higher amount of iatrogenic blood loss in IUGR-ELGANs (Figures 2, 3) (41). Transfusion volumes of 15–20 ml/kg per transfusion were the recommended volumes at that time (42). Due to the lack of robust evidence especially in the beginning of the study period, no standard transfusion threshold was defined in our institution. With the participation in the ETTNO trial (43) from 2011 onwards, infants received RBC transfusions depending on the randomization or (if not included in the RCT) according to the restrictive thresholds of the ETTNO protocol due to the evidence of the systematic review of Whyte and Kirpalani (44). We cannot distinguish whether the higher transfusion rates reflect the same intensity of laboratory diagnostics in a situation of lower total blood volume due to lower birth weight or whether the often more critical conditions urged the attending physician to maintain higher Hb levels in order to optimize circulatory stability and oxygen carrier capacity. Standard procedures for blood examinations at admission and third day of life were the same for all ELGANs during the whole study period.

This raises the question on a higher incidence of NEC and severe ROP that previously have been associated with RBC transfusions in observational studies (45). However, there was no difference in our study for the incidence of NEC and severe ROP or the need for intervention in ROP, although an increased *a priori* risk for NEC or severe ROP has been reported in preterm infants with IUGR (46, 47). A Cochrane meta-analysis as well as two recent RCTs on RBC transfusion thresholds did not show any significant association of death, major morbidities (NEC, ROP, and BPD), or impaired neurodevelopmental outcome in infants transfused according to restrictive vs. liberal transfusion thresholds (43, 44, 48, 49). An attractive alternative approach to avoid RBC transfusions not only by late cord clamping, reduction of iatrogenic blood loss (e.g., no routine coagulation screening in non-bleeding infants), and iron supplementation could be the use of recombinant human erythropoietin (rhEPO), recently shown to be effective in reducing transfusion needs in extreme low birth weight infants (50).

### Thrombocytopenia and Platelet Transfusions

In our study, IUGR-ELGANs had lower PLT at birth, but severe thrombocytopenia was rare. Besides the suppression of megakaryopoiesis by excessive erythropoiesis, abnormal villous vessels in placental dysfunction are discussed, causing microangiopathic sequestration and destruction of PLT (35). Notably, there was no difference in major hemorrhage when comparing IUGR-ELGANs with controls, and GA was the only independent risk factor in the regression analysis.

Infants with severe IUGR, however, received intriguingly more PLT transfusions during the first week of life (Figure 2B). As recently reviewed, thresholds for PLT transfusions in very low birth weight infants vary widely (51). During the study period, no clearly defined threshold for PLT transfusions existed in our neonatal intensive care unit (NICU). Depending on the general condition, transfusions of PLT were administered at PLT counts of 50 to 100/nl in a non-bleeding patient; therefore, most of them have been given in the intention to prevent major hemorrhage. We cannot conclude whether this contributed to the low incidence of major hemorrhage. The recent large RCT on PLT transfusions (PlaNeT2), however, showed a higher risk of death or major hemorrhage in the liberal threshold group <50/nl compared to a group receiving PLT transfusions at a restrictive threshold of <25/nl (14). In our study, a higher frequency and volume of PLT transfusions were associated with a higher mortality rate in IUGR-ELGANs, which is in line with the results of PlaNeT2 (14). To date, it remains to be elucidated whether ELGANs with severe IUGR may require individualized or personalized PLT transfusion thresholds if the coagulation screen (or PLT function) may suggest a higher bleeding risk as result of liver dysfunction due to highly stimulated erythropoiesis.

### Neutropenia and Infection

The hematopoietic shift toward excessive erythropoiesis is likely to cause the severe neutropenia in IUGR-ELGANs. Besides, neutropenia could also result from a placenta-derived factor that causes in pregnancy-induced hypertension *per se* a reduced production of neutrophils (52). Considering the high mortality rate and significant long-term morbidity (53), the question of the use of (initial) prophylactic antibiotic treatment or the implementation of antibiotic stewardship requires specific attention. In our study, 23/38 (60.5%) of all IUGR-ELGANs treated with antibiotics on the first day after birth did not exhibit elevated inflammatory parameters. However, 7 out of these 23 infants (30.4%) and further 8 out of 11 infants (72%) without initial antibiotic treatment after birth showed a secondary raise of inflammatory parameters. Due to the suppressed granulopoiesis, infection prevention control should eventually include antibiotic therapy in IUGR-ELGAN during the quantitative and functional recovery of granulopoiesis, despite the evidence that a prolonged initial empiric antibiotic treatment (≥5 days) may lead to a higher rate of LOI, NEC, BPD, ROP, and mortality (28, 54). However, whether IUGR-ELGANs exhibit a reduced cellular or humoral defense against bacterial infection during the recovery of granulopoiesis deserves further clinical and laboratory research.

The higher rate of chorioamnionitis in controls might reflect the cause of preterm birth in controls. Intriguingly, the postnatal risk for developing I-ENP was significantly elevated in IUGR-ELGANs (Table 5). In a binary logistic regression, IUGR remained the only independent variable for increased inflammatory markers during the first week of life. So far, it is unclear whether the inflammatory response is primarily associated with early-onset neutropenia (55, 56). It should be discussed whether the higher rate of I-ENP reflects not only initially impaired granulopoiesis, but also reduced cellular function in stress granulopoiesis in IUGR-ELGANs (Table 5) (57). Notably, on day 7, 62.7% of IUGR-ELGANs were treated with antibiotics compared to 24.5% of controls (Table 4, Supplementary Figure 2). However, there was also a significant association between severe IUGR and the incidence of at least one late inflammatory response. The German national surveillance system for nosocomial infection in very low birth-weight infants (NEO-KISS) clearly demonstrated correlation between the infants' birthweight and the incidence of infections (58). Furthermore, a subgroup analysis of a recent cohort study of the German Neonatal Network associated IUGR with early- and late-onset sepsis, as well as clinically suspected and culture-proven sepsis (59). Longer and repetitive antibiotic treatment was shown in previous studies (58) and our cohort. The implication on antibiotic stewardship and long-term morbidities is yet unclear.

### Morbidities and Mortality

The well-known association of IUGR and BPD (60, 61) is also found in our analysis of IUGR-ELGANs. The development of BPD was independently associated with GA, IUGR, and days on mechanical ventilation, but the volume of transfusions did not reach significance for the blood components or frequency of infections in binary logistic regression analysis. However, our cohort is likely too small to show the effect of PLT transfusion compared to the much more important risk factors such as GA, birthweight, or invasive ventilation (47). The latter might be a contributing factor, as the secondary outcome of PlaNeT2 showed a higher incidence of BPD in the group with liberal transfusion threshold. PLTs have an immunologic function and transfusions of adult PLT might cause a proinflammatory response in preterm infants (14). The pathophysiology could include the release of biological response modifiers from stored adult PLTs, claimed to worsen inflammatory injury and BPD (62, 63).

The population size in our subgroup of IUGR-ELGANs does not allow to associate the degree of aberrant hematopoietic findings (e.g., severe thrombocytopenia with a platelet number <50/nl) to the frequency of certain morbidities.

Our study showed a more than sevenfold higher mortality risk for IUGR ELGANs compared to controls comparable to former studies (60, 64). A subgroup analysis of the deceased IUGR infants did not show any association with infections or other life-threatening comorbidities such as severe abdominal complications (Supplementary Table 1). In line with a recent cohort study (65), we showed that a severe pulmonary morbidity occurred more often in IUGR-ELGANs and contributed to the early and late mortality in IUGR infants. Treatment of the later deceased infants in both groups was characterized by more intensive care with longer need of invasive ventilation and supplemental oxygen relative to days of survival, prolonged antibiotic treatment, and more RBC and PLT transfusions implicating the serious condition (Supplementary Table 1).

The retrospective character of this study bears the risk of uncertain causality, and the long study period can lead to bias due to changes in scientific knowledge and altered standard operating procedures. As this is a monocentric study, a further limitation is the low number of IUGR-ELGANs. However, our case group was carefully matched not only according to GA and sex, but also with regard to the year of neonatal care in order to consider eventual changes in clinical routine. Regarding the low number of cases, the 1:2 allocation strengthens the power of the statistical analysis and therefore provides substantial information on an especially vulnerable group of extreme preterm infants regarding the severe growth restriction.

In summary, the disturbed hematopoiesis in IUGR-ELGAN deserves particular attention. Infection prevention control should eventually include antibiotic treatment during the recovery period of granulopoiesis. Although rather restrictive transfusion thresholds and policies appear to be beneficial, this very vulnerable group of infants is not adequately represented in current RCTs on neonatal transfusions.

## Data Availability Statement

The raw data supporting the conclusions of this article will be made available by the authors, without undue reservation.

## Ethics Statement

The studies involving human participants were reviewed and approved by Institution Review Board, Charité - University Medicine, Berlin, Protocol No. EA2/053/18. Written informed consent from the participants' legal guardian/next of kin was not required to participate in this study in accordance with the national legislation and the institutional requirements.

## Author Contributions

CD and TM developed the study concept and design. NR and TM obtained, analyzed, interpreted the data, and wrote the first draft of the manuscript. CD contributed to interpretation of the results. CD and CB significantly contributed to the discussion and revised the first draft. All authors contributed to the article and approved the submitted version.

## Conflict of Interest

The authors declare that the research was conducted in the absence of any commercial or financial relationships that could be construed as a potential conflict of interest.

## Publisher's Note

All claims expressed in this article are solely those of the authors and do not necessarily represent those of their affiliated organizations, or those of the publisher, the editors and the reviewers. Any product that may be evaluated in this article, or claim that may be made by its manufacturer, is not guaranteed or endorsed by the publisher.

## Abbreviations

ANC: absolute neutrophil count
aPTT: activated partial thromboplastin time
BPD: bronchopulmonary dysplasia
CBC: complete blood count
CrP: C-reactive protein
ELGAN: extremely low gestational age newborn
EOI: early-onset infection
EPO: erythropoietin
FFP: fresh frozen plasma
GA: gestational age
I-ENP: infections within the early neonatal period
IL-6: interleukin-6
INR: international normalized ratio
IUGR: intrauterine growth restriction
IVH: intraventricular hemorrhage
LOI: late-onset infection
NEC: necrotizing enterocolitis
NICU: neonatal intensive care unit
NRBC: nucleated red blood cells
PAH: pulmonary arterial hypertension
PH: pulmonary hemorrhage
PLT: platelets
PPHN: persistent pulmonary hypertension
RCT: randomized controlled trial
ROP: retinopathy of prematurity
SIP: spontaneous intestinal perforation
VLBW: very low birthweight
WBC: white blood count.
