# Supplementary material for: Aberrant Hematopoiesis and Morbidity in Extremely Preterm Infants With Intrauterine Growth Restriction
Source: Front Pediatr. 2021 Nov 12;9:728607. doi: 10.3389/fped.2021.728607 (PMC8633541; doi:10.3389/fped.2021.728607)
Supplement: Supplementary file 1 [file Table_1.DOCX]

|  | Deceased infants IUGR (n=18) | Surviving infants IUGR (n=31) | p-value | Deceased infants controls (n=7) | Surviving infants control (n=91) | p-value | **p-value |
| --- | --- | --- | --- | --- | --- | --- | --- |
| Laboratory results at birth |  |  |  |  |  |  |  |
| NRBC (/nl) | 12.93 (2.84-39.44) | 10.7 (0.76 – 75.46) | .52 | 11.54 (1.48 – 37.96) | 3.14 (0.15 – 75.31) | .03 | <.001 |
| PLT (/nl) | 97 (52 - 179) | 116 (32 - 232) | <.05 | 263 (103 – 479) | 219 (58 – 427) | .44 | <.01 |
| WBC (/nl) | 4.28 (1.65 – 8.7) | 4.88 (2.12 – 14.41) | .25 | 18.33 (7.44 – 60.77) | 8.66 (3.18 – 63.82) | <.05 | <.001 |
| ANC (/nl) | 0.44 (0.05 – 2.43) | 0.54 (0.16 – 3.40) | .10 | 5211 (2686 – 40716) | 2449 (103 – 35101) | <.05 | <.001 |
| IL-6 (ng/l) | 20 (2 – 164.7) | 37 (2 – 304) | .54 | 107.5 (18.4 – 3802) | 64.6 (1.5 – 50000) | .30 | <.05 |
| CrP (mg/dl) | 0.3 (0.3 – 0.3) | 0.3 (0.03 – 3.3) | .84 | 9.4 (0.7 – 18.1) | 0.3 (0.1. – 19.4) | .09 | .10 |
| Complications |  |  |  |  |  |  |  |
| PH | 1 (5.6) | 1 (3.2) | 1.0 | 0 (0) | 2 (2.2) | 1.0 | 1.0 |
| IVH (all grades) | 6 (33.3) | 1 (3.2) | <.01 | 3 (42.9) | 11 (12.1) | .58 | .67 |
| Severe IVH | 4 (22.2) | 1 (3.2) | .54 | 2 (28.6) | 3 (3.3) | <.05 | 1.0 |
| PPHN | 7 (38.9) | 4 (12.9) | .72 | 2 (28.6) | 1 (1.1) | <.05 | 1.0 |
| Late PPHN | 4/4 (100) | 6/31 (19.4) | <.01 | 0 (0) | 8/91 (8.8) | - | .19 |
| BPD | 6 (33.3) | 30 (96.8) | <.001 | 4 (57.1) | 57 (62.6) | 1.0 | .38 |
| BPD, death before 28  days = missing | 6/6 (100) | 30 (96.8) | 1.0 | 4/4 (100) | 57 (62.6) | .29 | - |
| Severe abdominal complications | 4 (22.2) | 6 (19.4) | 1.0 | 4 (57.1) | 4 (4.4) | <.01 | .16 |
| NEC | 2 (11.1) | 1 (3.2) | .55 | 2 (28.6) | 2 (2.2) | <.05 | .55 |
| SIP | 1 (5.6) | 3 (9.7) | 1.0 | 1 (14.3) | 1 (1.1) | .14 | .49 |
| Measures and treatment |  |  |  |  |  |  |  |
| Surfactant-therapy | 18 (100) | 29 (93.5) | .52 | 7 (100) | 78 (85.7) | .59 | - |
| Days on invasive ventilation |  |  |  |  |  |  | .53 |
| absolute | 20 (2 – 112) | 23 (0 – 72) | .69 | 15 (10 – 94) | 4 (0 – 95) | <.01 |  |
| relative to days until  death or discharge | 100 (18.9 – 100) | 22.3 (0 – 63.7) | <.001 | 97.9 (53.6 – 100) | 5.9 (0 – 87.2) | <.001 | .22 |
| Days on supplemental oxygen |  |  |  |  |  |  |  |
| absolute | 21 (2 – 263) | 80 (1 – 204) | <.01 | 23 (10 – 96) | 46 (0 – 160) | .26 | .53 |
| relative to days until  death or discharge | 100 (96.9 – 100) | 78.4 (1.2 – 100) | <.001 | 100 (82.1 – 100) | 55. (0 – 100) | <.01 | .80 |
| Days on antibiotic treatment |  |  |  |  |  |  |  |
| absolute | 13 (0 – 72) | 17 (4 – 92) | .25 | 11 (7 – 53) | 8 (0 – 88) | .07 | .95 |
| relative to days until death  or discharge | 92.4 (0 – 133.3) | 18.2 (4.1 – 80) | <.001 | 64.1 (22.5 – 91.7) | 9.4 (0 – 73.1) | <.001 | .13 |
| Abdominal surgery | 2 (11.1) | 6 (19.4) | .70 | 3 (42.9) | 4 (4.4) | <.01 | .11 |
| Transfusions |  |  |  |  |  |  |  |
| RBCs | 57.3 (18.2 – 164.5) | 19.5 (0 – 78.6) | <.01 | 20.5 (14.9 – 77.1) | 0 (0 – 21.1) | <.05 | <.05 |
| PLTs | 34.9 (0 – 102.8) | 16.0 (0 – 69.8) | <.01 | 0 (0 – 81.4) | 0 (0 – 21.1) | .31 | <.01 |
| FFP | 22.1 (0 – 96.8) | 19.2 (0 – 85.7) | .12 | 14.9 (0 – 71.4) | 14.7 (0 – 38.9) | .95 | <.05 |
