# Supplementary material for: Aberrant Hematopoiesis and Morbidity in Extremely Preterm Infants With Intrauterine Growth Restriction
Source: Front Pediatr. 2021 Nov 12;9:728607. doi: 10.3389/fped.2021.728607 (PMC8633541; doi:10.3389/fped.2021.728607)

Supplemental Figure 1:
This figure illustrates transfusion treatment (red blood cell transfusions (red), platelet transfusions (green) and fresh frozen plasma (yellow)) during the first week of life. The left diagram shows the control group, the diagram the IUGR group. Days of life are plotted on the x-axis, each row of the y-axis represents a child, sorted by severity of disease (time of death, incidence of late onset sepsis, infections treated with antibiotic reserve, no antibiotic treatment) from lowest in the first line to severest condition in bottom line. The length of the bar reflects the number of transfusions, ranging from 1 to 3 per patient. Pink bars indicate diagnosis of a hemorrhagic event. Gray bars indicate death of the infant. Infants beneath the black horizontal line marked with arrowheads died during hospital stay.

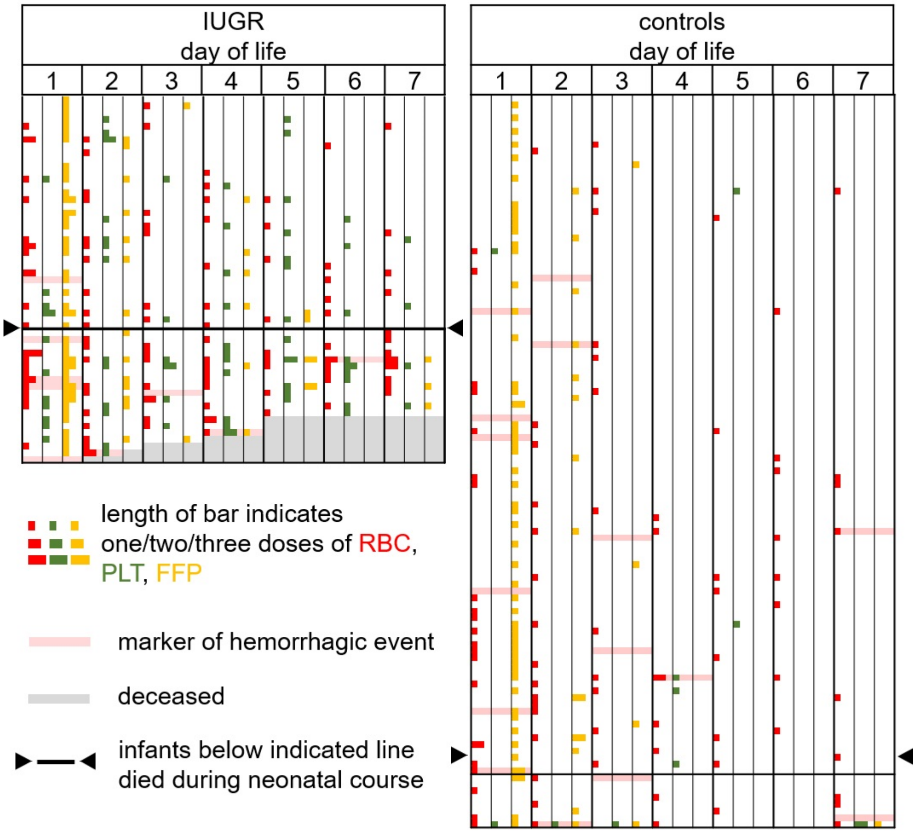

Supplement: Supplementary file 2 [file Image_1.pdf]
