# Supplementary material for: Aberrant Hematopoiesis and Morbidity in Extremely Preterm Infants With Intrauterine Growth Restriction
Source: Front Pediatr. 2021 Nov 12;9:728607. doi: 10.3389/fped.2021.728607 (PMC8633541; doi:10.3389/fped.2021.728607)

Supplemental Figure 2:

Illustration of infections and antibiotic treatment in the first 28 days of life. The left diagram shows the IUGR group, the right one the control group. Days of life are plotted on the x-axis, each row of the y-axis represents one infant, sorted by severity of disease as described in Supplemental Figure 1 from lowest in the first line to severest condition in bottom line. Red bar: first-line antibiotic treatment (ampicillin plus gentamicin), dark-red bar: extended first line antibiotic treatment (piperacillin/tazobactam or cephalosporine), brown bar: second line antibiotics (vancomycin and/or meropenem), black bar: deceased. Hatched areas: treatment due to elevated inflammation parameters, plain areas: treatment at discretion of attending neonatologist due to suspected sepsis. First column day 0: pink marker indicates chorioamnionitis. Infants beneath the black horizontal line marked with arrowheads died during hospital stay.

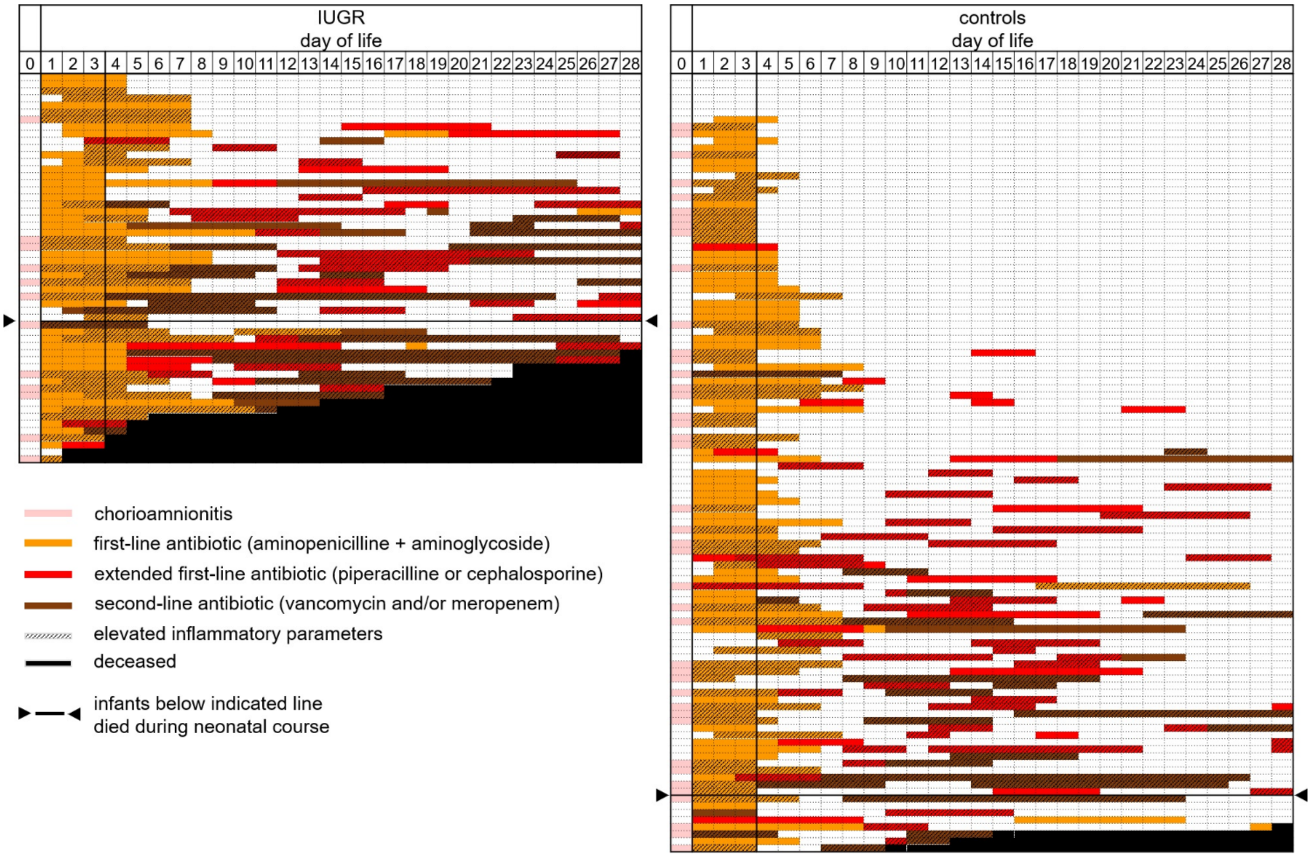

Supplement: Supplementary file 3 [file Image_2.pdf]
